# Study of different doses of zinc oxide nanoparticles by intraperitoneal and gavage methods on testicular tissue in Wistar rats: An experimental study

**DOI:** 10.18502/ijrm.v21i6.13637

**Published:** 2023-07-24

**Authors:** Saemeh Rezaei Larijani, Seyed Mohammad Hosseini, Behrang Ekrami

**Affiliations:** ^1^Department of Pathology, Babol Branch, Islamic Azad University, Babol, Iran.; ^2^Department of Animal Science, Chaloos Branch, Islamic Azad University, Chaloos, Iran.

**Keywords:** Zinc oxide, Testis, Nanoparticle, Oxidative stress, Testosterone.

## Abstract

**Background:**

Zinc oxide nanoparticles (ZnO-NPS) are widely used inhuman life; however, they do have side effects on human health.

**Objective:**

This study aimed to evaluatethedifferent doses of ZnO-NPS on testicular tissue.

**Materials and Methods:**

35 male Wistar rats(10-12 wk, 220 ± 20gr) were divided into 7 groups of 5, including the control group (gavaged distilled water daily), sham group (received intraperitoneal doses of distilled water twice a week). The group receivedintraperitoneal ZnO-NPS (25, 50, and 100 mg/kg body weight, twice a week), and gavage (150 and 200 mg/kg body weight daily). All stages of the test were performed in 4 wk then serum testosterone and tissue malondialdehyde, and ferric reducing antioxidant power levels were measured, also testes histopathological evaluation was performed.

**Results:**

Our results showed that a reduced cell population of spermatozoa was observed in the group that received 25 mg/kg ZnO-NPS, while a reduced cell population of spermatozoa, edema, hyperemia, and vacuolar degeneration were observed in the group that received 50 and 100 mg/kg ZnO-NPS. The maximum amount of lesions were observed in the dose of 200 mg/kg. The highest amount of ferric reducing antioxidant power and testosterone levels were observed in the control group. Also, a 100 mg/kg intraperitoneal dose of ZnO-NPS and 150 mg/kg oral dose of ZnO-NPS were suitable doses to create a model of male genital lesions.

**Conclusion:**

Nanoparticles are harmful factors for the reproductive system and consequently affect infertility, which requires the toxicity of the concentration of these nanoparticles to be evaluated and controlled.

## 1. Introduction

Humans are exposed to a variety of environmental pollutants throughout their lives. Nowadays, due to the increase in the number of chemical industries, the environment is polluted with various toxic substances. Concerns about the harmful effects of heavy metals have increased in recent years. Therefore, recognizing the impact of heavy metals on human health is very important (1). Zinc oxide (ZnO) is used as a white powder for paints, cosmetics, glass, and inks. Also, it is one of the most widely used materials in various industrial fields (2). According to the scientific community's definition, a nanoparticle is a particle with dimensions between 1 and 100 nanometers whose general properties change as the particles get constricted. In nanotechnology, the first effect of reducing particle size is to increase the particle level.

When the surface-to-volume ratio of nanoparticles is increased, the physical properties of the particles are influenced more by atoms on the surface than by atoms within the particle volume. This feature enhances nanoparticle reactivity (3). There is also a general belief that some of these metals are necessary for organisms to perform their biochemical and physiological functions, but become toxic when their concentration exceeds their threshold (4).

Because zinc oxide nanoparticles (ZnO-NPS) are widely used in industrial settings, it is advisable to expect that the human body will be exposed to nanoparticles in a variety of ways, including ingestion, inhalation, intravenous injection, and skin penetration (5). It has been shown that ZnO-NPS in mammalian cells can disturb the balance of zinc ions and cause mitochondrial and lysosomal damage by producing oxygen free radicals and ultimately leading to apoptosis (6). Infertility has been a significant problem for couples in recent years. Statistical studies have shown that about 50% of these cases are related to the malefactor. Any abnormality in the structure of the testicles adversely affects sperm. In addition, it harms the fetus (7-9). Many researchers have examined the potential side effects of nanoparticles and announced that long-term encounter to environmental contamination such as zinc can lead to unfavorable wellbeing and detrimental effects on sexual performance (10).

This study aims to investigate the effect of different doses of ZnO-NPS on the reproductive system of Wistar rats.

## 2. Materials and Methods

### Materials

ZnO-NPS with a size of 25-50 nm, which was almost spherical in the form of white milk crystal were prepared from the Danesh Bonyan Company in the University of Tehran, Tehran, Iran. The nanoparticle powder was weighed under the hood based on the weight of the rats and kept in 5 ml Eppendorf tube (Germany). 0.9% typical saline solution was used for dilution.

### Experimental animals

This experimental study was performed on 35 male Wistar rats within the age range of 10-12 wk and weighed approximately 220 ± 20 gr when purchased from the Pasteur Institute of Iran, North Research Center Iran in 2018. Rats were kept in the animal house of the Islamic Azad University of Babol Branch, Babol, Iran in a 12 hr light/dark cycle with a temperature of 23 ± 2, relative humidity of 5 ± 5, and free access to water and food (11).

### Study design

Rats were randomly divided into 7 groups (n = 5/each) as follows:

Group 1 (Control): fed daily with normal water and food without any additives or drugs in the diet.

Group 2 (Sham): received 0.5 ml of 0.9% typical saline solution intraperitoneally (i.p.) twice a week.

Group 3-5: received ZnO-NPS by i.p. injection (25, 50, and 100 mg per kg body weight, twice a week)

Group 6, 7: received ZnO-NPS by gavage (150 and 200 mg per kg body weight, daily).

All these steps were performed simultaneously for 4 wk (12).

### Bodyweight and hormonal assay

An i.p. mixture of ketamine (60 mg/kg body weight) (Bremer Pharma GmbH, Germany) and xylazine (20 mg/kg body weight) (Alfasan Woerden, Holland) was used to anesthetize the rats after 4 wk, and blood samples (3-5 cc) from the heart were collected. The Monobind Kit (Monobind, INC. USA) methodology was used to quantify serum testosterone levels in all rats.

### Evaluation of malondialdehyde (MDA)

Homogenizers were created by homogenizing 0.5 gr of testicular tissue with 5 ml of saline phosphate buffer (Polytron, Heidolph RZR 1, Germany). The suspension was centrifuged at 12,000 gr for 15 min at 15 C (Heraeus, Germany: Biofuge Primo R). In the supernatant, MDA was measured. The samples were assessed directly. According to the business, this metric was tested using Zell Bio diagnostic kits (Zell Bio, Germany).

### Evaluation of ferric reducing antioxidant power (FRAP)

According to this approach, the emergence of an aqueous compound is caused by the development of a complex between Fe ${}^{2+}$ and tripyridyltriazine (Sigma-Aldrich GmbH, Steinheim, Germany). Consider the following FRAP worker reagent (300 mmol/L acetate buffer, pH 3.6, 10 mmol/l tripyridyltriazine at 40 mmol/l hydrochloric acids, and 20 mmol/l FeCl ${}_{3}$ 10: 1: 1): Mix 50 μl of the sample supernatant in the sole empty reagent. It was then read at 593 nm after 4 min of incubation at 37 C. FeSO ${}_{4}$ 7H ${}_{2}$ solution in the range of 100-1000 μmol/l was also used for calibration.

### Histopathological examination

All histopathological examinations of testicular tissues were performed using optical microscopy (Olympus CX21, Japan). After completing different stages of perfusion, the testicular tissue is fixed in neutral 10% buffer, cut into 5 μm thick pieces, and after processing the tissue embedded in paraffin blocks, it is installed on glass slides. Hematoxylin and eosin were used to stain sections (11).

Each testis histopathological lesions were given a score of 0-3 for decreased cell population, edema, vacuolar degeneration, and hyperemia (mild lesions with (+), moderate lesions with (++), severe lesions with (+++), and no lesions were shown as (-)) in compliance with the articles for statistical analysis (13, 14).

### Ethical considerations

The Animal Ethics Committee of Islamic Azad University, Babol Branch, Babol, Iran approved the experimental design (Code: IR.IAU.BABOL.REC.1397.009). The ARRIVE guidelines 2.0 was also considered and followed (15).

### Statistical analysis

Quantitative variables were presented as mean ± SD. The MDA, FRAP, and Testosterone levels were compared by one-way ANOVA, and Duncan was used as post hoc test. P < 0.05 was considered to be statistically significant. The Kolmogorov-Smirnov test was used to evaluate the normality of the quantitative data. The data were analyzed using IBM Corp. Released in 2019. IBM SPSS Statistics for Windows, Version 26.0. Armonk, NY: IBM Corp.

## 3. Results

### Histopathological findings

The histopathological finding shows that the normal tissuecondition was present in the testicular tissues of the control group and sham group, and no lesions were observed. In testicular tissue in the group of 25 mg/kg, a decrease in cell population was marked, so that the number of spermatozoa in the lumen of the tubes decreased.

**Figure 1:**
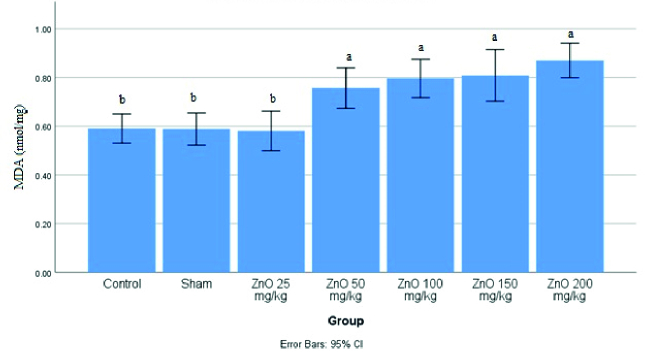
Shows the normal structural conditions in control and sham groups, Sertoli cells (S), spermatogonium (SP), primary spermatocyte (PSC), spermatid (ST), spermatozoon (SZ), Leydig (L), tissue lesions in treatment groups, cell population reduction, edema (right arrow), vacuolar degeneration (up arrow), left arrow (hyperemia), H&E staining, × 10, × 40 magnification scale bar; 100 µm.

In testicular tissue in the 50 mg/kg group, a decrease in the spermatozoa population in the lumen, interstitial edema, and vacuolar degeneration in Sertoli cells and spermatogonia were observed. In the 100 mg/kg group, vacuolar decline was observed in Sertoli cells and spermatogonia, interstitial edema, decreased spermatozoa cell population, and hyperemia. In the 150 mg/kg group, vacuolar degeneration in Sertoli cells and spermatogonia, interstitial edema, decreased spermatozoa cell population, and hyperemia were observed. In the group of 200 mg/kg, vacuolar decline was observed in Sertoli cells and spermatogonia; interstitial edema, decreased spermatozoa cell population, and hyperemia were also observed (Figure 1).

### Oxidative stress (OS) evaluation

Although the selected gavage doses were higher than the injectable doses, the injected doses had more destructive effects on testicular tissue. The severity of lesions was lower at 150 mg/kg (gavage) than at 100 mg/kg (i.p. injection). Drugs administered in the i.p. have more destructive effects due to their excellent absorption than drugs administered in the gastrointestinal tract. However, at a dose of 200 mg/kg (gavage), a severe maximum decrease was observed in the spermatozoa cell population, moderate edema, mild hyperemia, and vacuolar degeneration (Table I, Figure 1).

**Figure 2:**
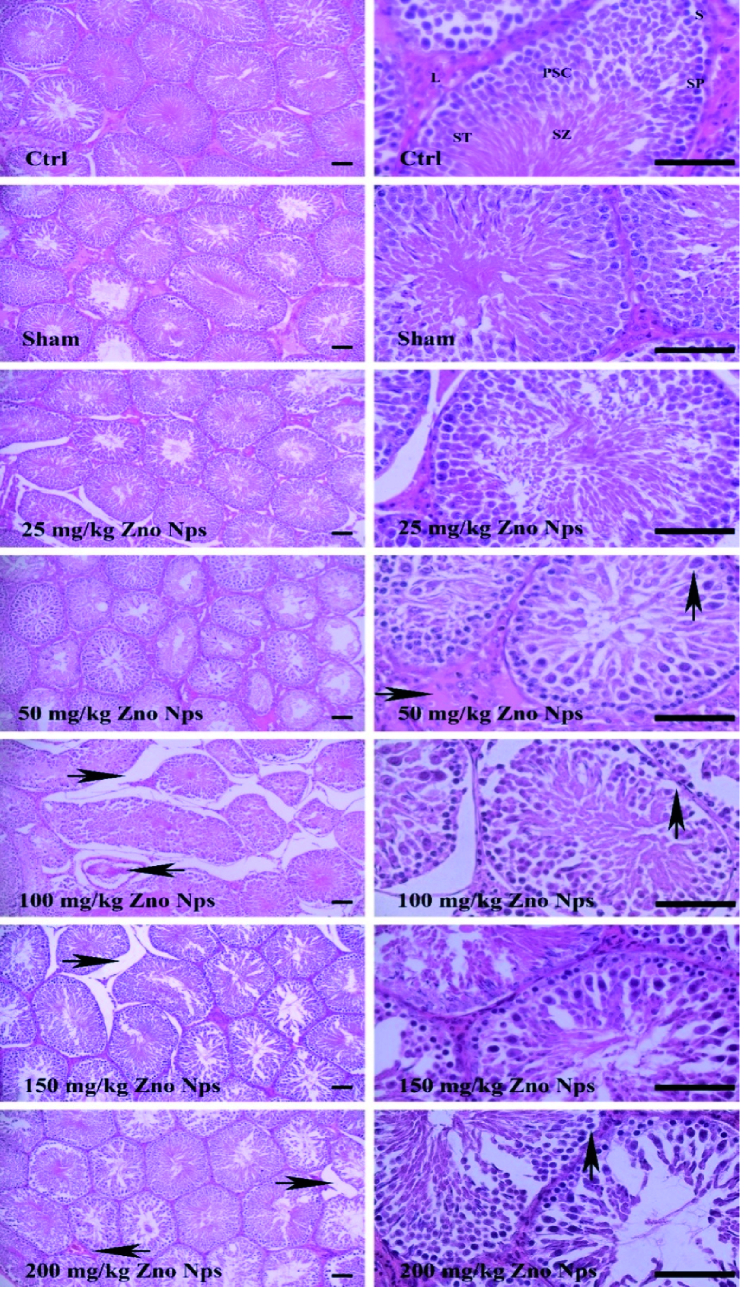
Levels of malondialdehyde (MDA) in the testis of male rats treated with different doses of zinc oxide nanoparticles (ZnO-NPS). Significant difference between the groups shown with different letters (a and b) (p < 0.05).

Figure 2 shows that the highest amount of MDA was in the group receiving ZnO-NPS at a dose of 200 mg/kg. However, with increasing doses, the amount of MDA had increased significantly dose-dependent (p < 0.05). Nevertheless, it was not significantly different (p < 0.0001) from other doses.

**Figure 3:**
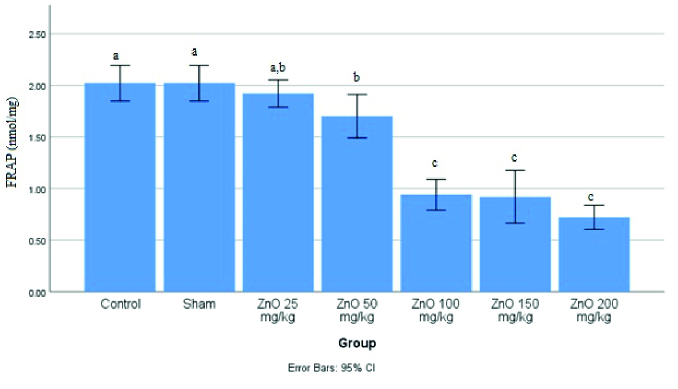
Levels of ferric reducing antioxidant power (FRAP) in the testis of male rats treated with different doses of ZnO-NPS. Significant difference between the groups shown with different letters (a, b, and c).

As shown in figure 3, the highest amount of FRAP was observed in the control and sham groups, significantly different from other groups. Also, the ZnO-NPS 50 mg/kg group showed a significant difference between the treatment groups (p < 0.0001).

**Figure 4:**
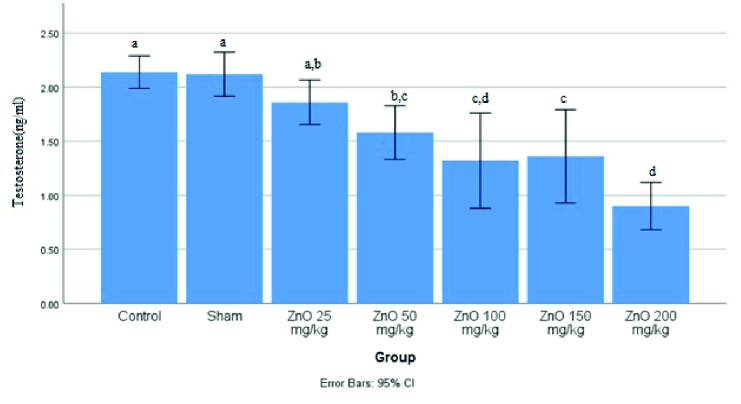
Levels of testosterone of male rats treated with different doses of ZnO-NPS. Significant difference between the groups shown with different letters (a, b, c, and d).

Figure 4 shows that the highest testosterone level was in the control group, which did not show a significant difference with the sham and ZnO-NPS 25 mg/kg groups. Yet, a significant difference was observed between the other groups (p < 0.0001). Also, the lowest testosterone level was found to be in the ZnO-NPS 200 mg/kg group.

**Table 1:** Histopathological evaluation of testicular tissue of rats treated with different doses of zinc oxide nanoparticles (ZnO-NPS)


**Groups **	**Decreased cell population**	**Edema**	**Vacuole degeneration**	**Hyperemia**
**Control**	**-**	**-**	**-**	-
**Sham**	**-**	**-**	**-**	-
**25 mg/kg**	**+**	**-**	**-**	-
**50 mg/kg**	**++**	**+**	**+**	+
**100 mg/kg**	**++**	**++**	**+**	++
**150 mg/kg**	**++**	**+**	**+**	+
**200 mg/kg**	**+++**	**++**	**+**	+

## 4. Discussion

Due to its ultraviolet-blocking properties, ZnO-NPS are also used in sunscreens, cosmetics, facial creams, ointments, lotions, and bottle coatings. They are useful in dietary supplements, food additives, pharmaceuticals, and sunscreens for human and livestock consumption. A wide range of industries use NPS, which can cause contamination in the environment and indirect human exposure, but on the other hand, these NPS are increasingly used in biological and medical applications, including cancer diagnosis and therapy, which expose people directly to them. Heavy metals such as zinc can have destructive effects on different organs and systems over short- and long-term exposures (7). In the present study, a histopathological examination of the testis tissue of the control and sham groups revealed no microscopic lesions in contrast to those groups that received ZnO-NPS (groups 3-7). These lesions include decreased cell population, edema, vacuole degeneration, and hyperemia. It was found that testis injected with ZnO-NPS had a decrease in germinal cells, hyalinization of the luminal substance, with vacuolar degeneration of the Sertoli cells and germinal epithelium, as well as interstitial edema (16).

Gold has been shown to reduce sperm motility, and the presence of gold in semen may increase the likelihood of sperm toxicity and cause male infertility (17-19). The development of ZnO and ZnO-NPS on the gill tissue pathology of rainbow trout was investigated. This study shows that ZnO-NPS at a concentration of more than 50 mg/kg can cause significant changes in sperm quality and quantity (20). Cadmium reduced spermatogenesis maturity and major quantitative testicular parameters. Consumption of cadmium chloride destroys all testicular germ cells due to its strong oxidizing properties, which have destructive effects on proliferating cells (21, 22).

Gold and silver nanoparticles have been shown to cause reproductive toxicity by damaging the function of sperm, somatic cells, and mammalian gametes, causing irreversible effects by deposition in the reproductive glands. Of course, it should be noted that the degree of toxicity of nanoparticles depends on the dose, exposure time, and tissue studied with different consequences and effects (16, 22). Lesions such as necrosis, “hyperemia, hyaline casts, inflammatory cell infiltration, glomerular proliferation, and fibrosis were observed during i.p. injection of different doses of ZnO-NPS into renal tissue. And most lesions occurred in the 200 mg/kg group" (21).

According to the previous studies, based on the present study results, receiving ZnO-NPS led to a significant reduction in the cell population of spermatozoa and edema, hyperemia, and vacuolar degeneration (8, 18, 20, 22).

Silver nanoparticles were studied for their effects on sperm cell count, Leydig cell count, testosterone, luteinizing hormone, follicle-stimulating hormone, and sex hormone levels. Their research found that the number of Leydig cells in the study groups was significantly reduced (23). By examining the effect of titanium oxide nanoparticles on the fertility of rats, they showed that these nanoparticles, by inducing OS pathways, severely reduced the vitality of sperm, Leydig cells, and the expression of some genes (18).

Examination of ZnO-NPS on testicular function in male rats showed significantly reduced testosterone levels (16). Another study on ZnO-NPS in low doses on 36 Wistar rats shows a significant increase in the testosterone levels in the blood serum of rats injected with ZnO-NPS rather than controls (24).

In the present study, we had a significant decrease in testosterone at a dose of 200 mg/kg. So, we concluded that it has a positive effect at doses below 50 mg/kg, and at higher doses, it has a side effect on the body. According to another study, cadmium elevated MDA in various organs, including the liver, kidney, brain, and testis (25, 26). Silver nanoparticles can damage sperm membranes, penetrate cells, and subsequently increase free radicals, including reactive oxygen species, causing lipid peroxidation of the membrane resulting in loss of motility and viability and damaging sperm membranes and flagella structure. And eventually, leading to sperm motility and morphological disorders (21). The ionic mechanism and OS are used by lead metal to generate toxicity in living cells. According to studies, OS in living cells is generated by a mismatch between the synthesis of free radicals and the growth of antioxidants to detoxify or repair the damage caused by reactive mediators (16). Lipid peroxidation occurs when free radicals gain electrons from lipid molecules within the cell membrane (21).

“Reactive oxygen species can cause structural damage to cells, proteins, nucleic acids, membranes, and lipids" at high concentrations, resulting in a stressed state at the cellular level (27). “The capacity of lead metal ions to substitute for other divalent cations such as Ca ${}^{2+,}$ Mg ${}^{2+}$ , and Fe ${}^{2+}$ , and monovalent cations such as Na+ is the fundamental ionic mechanism of lead poisoning," which disrupts the cell's metabolic processes. Lead toxicity has an impact on cell adhesion, “intracellular and intercellular communication, protein folding, maturation, apoptosis, ion transport, enzyme control, and neurotransmitter release" (10). By entering the mitochondria and causing physical damage that leads to OS, nanoparticles induce OS reactions, cause cell damage, and increase inflammation by activating related genes (28). The results of studies in this field showed that Getting exposed to ZnO-NPS might cause gene injury due to lipid peroxidation and OS in epidermal cells (29, 30). Additionally, studies have found that Zn ${}^{2+}$ released from ZnO plays a critical part in nanotoxicity (25). Likewise, the findings of the previous research were similar to those of the current investigation.

## 5. Conclusion

Although metal has many positive effects in low doses and plays an essential role in metabolism, it can be toxic at higher doses. Our study showed that high doses cause destructive histopathological effects, including edema, hyperemia, decreased cell population, and vacuolar degeneration in testicular tissue. It seems that the development of i.p. administration due to more absorption has a more significant effect than the gavage method, and 100 mg/kg i.p. dose and 150 mg/kg oral dose are suitable doses for creating a model of male genital lesions. Also, due to the formation of lesions at a dose of 50 mg/kg, nanoparticles should be used with more caution. Therefore, these particles are harmful factors for the reproductive system and consequently affect infertility. Therefore, it is necessary to evaluate and control the toxicity of the concentration of these nanoparticles.

##  Conflict of Interest

The authors declare that there is no conflict of interest.
